# Optimal dosage of cefmetazole for intraoperative antimicrobial prophylaxis in patients undergoing surgery for colorectal cancer

**DOI:** 10.1186/s40780-016-0071-6

**Published:** 2017-01-07

**Authors:** Atsushi Tomizawa, Takatoshi Nakamura, Toshiaki Komatsu, Hiroshi Inano, Rumiko Kondo, Masahiko Watanabe, Koichiro Atsuda

**Affiliations:** 1 Department of Pharmacy, Kitasato University Hospital, 1-15-1 Kitasato, Minami-ku, Sagamihara, Kanagawa 252-0374 Japan; 2 Department of Surgery, Kitasato University School of Medicine, 1-15-1 Kitasato, Minami-ku, Sagamihara, Kanagawa 252-0374 Japan

**Keywords:** Cefmetazole, Colorectal surgery, Antimicrobial prophylaxis, Population pharmacokinetics

## Abstract

**Background:**

Few studies have reported the dosage of cefmetazole (CMZ) for intraoperative antimicrobial prophylaxis in patients underwent surgery for colorectal cancer. We therefore examined the optimal intraoperative dosage of CMZ according to pharmacokinetic/pharmacodynamic (PK/PD) theory in patients who undergoing surgery for colorectal cancer.

**Methods:**

The study group comprised 23 patients with colorectal cancer who underwent surgery, using CMZ as antimicrobial treatment to prevent postoperative infection. CMZ was administered intravenously within 60 min before surgery. PK/PD analysis was performed by population pharmacokinetic analysis and Monte-Carlo simulation.

**Results:**

The final population pharmacokinetic parameters of CMZ were as follows: CL_CMZ_ = 0.0704 × creatinine clearance (Ccr) and Vd_CMZ_ = 0.163 × body weight (Bw). In patients with a Ccr of ≥90 to <130 mL/min, the probability of achieving concentrations exceeding MIC was 52.9 to 82.2% at 2 h after the initial dose and less than 20% at 3 h after the initial dose.

**Conclusions:**

Additional doses of CMZ should be given every 2 h in patients with a Ccr of ≥90 to <130 mL/min, every 3 h in those with a Ccr of ≥50 to <90 mL/min, and every 4 to 5 h in those with a Ccr of ≥10 to <50 mL/min.

## Background

Cefmetazole (CMZ) is a cephamycin’s antibiotics developed in Japan that has high antibacterial activity against gram-negative and anaerobic bacteria. It is widely used for antimicrobial prophylaxis (AMP) in patients undergoing lower gastrointestinal surgery [1].

Treatment schedules for AMP have been based on the Centers for Disease Control and Prevention guidelines [2], the recommendations of the Surgical Infection Prevention Guideline Writers Workgroup meeting [3], and recent collaborative guidelines issued by the American Society of Health-System Pharmacists, the Infectious Diseases Society of America, the Surgical Infection Society, and the Society for Healthcare Epidemiology of America [4]. A general consensus has also been reached in Japan. However, very few studies have evaluated the pharmacokinetics of CMZ during surgery for colorectal cancer and reported the optimal intraoperative treatment schedule for CMZ, including the timing of additional doses.

We studied the pharmacokinetics of CMZ during surgery in patients with colorectal cancer to determine the optimal dosage of CMZ on the basis of pharmacokinetics and pharmacodynamics.

## Methods

### Data source

The study group comprised 23 patients who underwent surgery for colorectal cancer and received CMZ for AMP between November 2008 and December 2010. Patients who underwent emergency surgery, those with ileus, and those who were receiving dialysis were excluded.

As for the treatment schedule, 1 g of CMZ was intravenously administered over the course of 5 to 10 min after the induction of anesthesia and within 60 min before the surgical incision. Subsequently, 1 g of CMZ was additionally given every 3 h. In principle, blood samples were collected at the start of surgery, on completion of the anastomosis, immediately before additional doses of AMP, and after abdominal closure.

### Assay of cefmetazole concentrations

Serum CMZ concentrations were measured by high-performance liquid chromatography (HPLC). After the completion of surgery, blood samples were centrifuged at 3000 rpm for 10 min, and the serum supernatant was preserved by freezing at −80 °C until assay. At the time of assay, 200 μL of serum was combined with 90 μL of a deproteinizing agent (1 M HClO_4_), and the mixture was centrifuged at 1000 rpm and 4 °C for 5 min. The obtained supernatant was filtered through a 0.45-μm syringe filter, and 50 μL of the filtrate was injected into a chromatograph. The HPLC column temperature was 25 °C, with an ultraviolet absorption wavelength of 256 nm. The mobile phase was prepared by combining 800 mL of 50 mM phosphate buffer (pH 4.5) with 200 mL of acetonitrile. The detection limit was 0.5 μg/mL.

### Pharmacokinetics model

Population pharmacokinetic analysis of CMZ was performed with the use of a nonlinear mixed effect model (NONMEM) program (version VI, level 1.0). For the pharmacokinetic model, we used predictions for population pharmacokinetics (PREDPP) subroutines with a linear one-compartment model (ADVAN 1 and TRANS 2) to estimate the pharmacokinetic parameters of the volume of distribution (Vd_CMZ_) and clearance (CL_CMZ_).

The inter-individual variability of the pharmacokinetic parameters was assessed using an exponential error model according to the following eq (1):

1 $$ {P}_j=P\times \exp \left({\upeta}_j\right) $$

Where *P*_*j*_ is parameter value of the **j**-th subject, *P* is the estimated population mean, and η_*i*_ is a random variable with a mean of 0 and a variance of ω^2^.

The intra-individual variability of the parameters was assessed using a proportional error model according to the following eq (2):

2 $$ {\mathrm{C}}_{ij}={\mathrm{C}}_{\mathrm{pred},ij}\times \left(1+{\upvarepsilon}_{ij}\right) $$

Where C_*ij*_ and C_pred*ij*_ denotes observed and predicted concentrations for the j-th subject at *i*-th time, and εis a random intra-individual error which is normally distributed with mean 0 and variance σ ^2^.

### Covariate analysis

The covariates of patients were performed for their influence on CMZ pharmacokinetic parameters as followed; age (Age), gender (Gender), body weight (Bw), clinical pathological stage (Stage), serum creatinine (Scr), creatinine clearance (Ccr), serum albumin (Alb), and operative procedure (Procedure). Operative procedures were divided into open surgery and laparoscopic surgery. Ccr was calculated using the Cockcroft-Gault equation.

The influence of continuous covariates on the pharmacokinetic parameter was modeled according to the following eqs (3, 4):

3 $$ \mathrm{P}=\theta p+\theta \mathrm{c}\times \left(\mathrm{covariance}\right) $$

4 $$ \mathrm{P}=\theta p\times \theta {\mathrm{c}}^{\left(\mathrm{covariance}\right)} $$

The significance of the influence of covariates was evaluated by the change of −2 log likelihood (the minimum value of the objective function: OBJ).

Statistical significance was indicated by a p value of <0.01. Only covariates providing a significant change in the OBJ were included in the full model and were then tested in a backward deletion step, with statistical significance indicated by a p value of <0.001. The ability of the final population pharmacokinetic model to describe adequately the observed data was evaluated using visual predictive values.

### Model evaluation

Actual serum CMZ concentrations in individual patients (Cp), predicted concentrations based on population parameters (PRED), and estimated individual predicted concentration calculated by Bayesian fitting (IPRED) were plotted to derive regression equations. Weighted residual values for Cp and PRED were plotted to evaluate the accuracy of serum concentrations estimated by the final model.

Parameter precision and model stability were estimated for the final model by the bootstrap method [5].200 bootstrap samples were reconstructed, and the final model was determined by the　200 bootstrap samples repeatedly tested. The mean and standard error (S.E.) for each estimated parameters calculated normally were compared with those obtained from the original data set.

### Evaluation of optimal dosage

A Monte-Carlo simulation [6] was performed 1000 times with the estimated and dispersion values of the population pharmacokinetic parameters, using Microsoft Excel 2010^®^. Estimated serum CMZ concentrations were calculated after 2, 3, 4, 5, and 6 h. On the basis of the minimum inhibitory concentration (MIC) distribution of *Bacteroides fragilis*, the probability of achieving serum CMZ concentrations above the MIC_80_ : MIC attainment rate, was calculated. As for the MIC distribution of *Bacteroides fragilis* for CMZ, a Japanese surveillance report of the antimicrobial susceptibility of clinical isolates of anaerobic bacteria in 2004 was used.

## Results

**Fig. 1:**
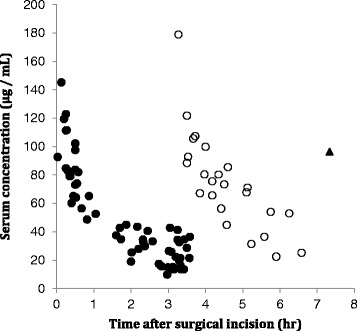
Observed cefmetazole serum concentration from 23 patients. The plots were showed after the first dose (●), the second dose (○), and the third dose (▲)

Table 1 shows the demographic characteristics of the patients. Serum concentrations were measured at a total of 86 points. The time course of serum CMZ concentrations is shown in Fig. 1. Ccr was a covariate that significantly influenced CL_CMZ_, and Bw was a covariate that significantly influenced Vd_CMZ_ (Table 2). These factors were integrated into the full model, which was compared with a reduced model. Consequently, the final CMZ population pharmacokinetic estimates were CL_CMZ_ = 0.0704 × Ccr and Vd_CMZ_ = 0.163 × Bw. The calculated interindividual variability (CV%) was 21.0% for CL_CMZ_ and 8.4% for Vd_CMZ_, and the residual variability was 13.5% (Table 3).

**Table 1:** Demographic characteristics of the patients

Parameter	Numbers	Mean ± SD	Range
Gender (male/female)	18 / 5		
Cancer (colon / rectum)	14 / 9		
Stage (I/II/III/IV)	10 / 6 / 6 / 1		
Procedure (lap^a^ / open)	13 / 10		
Age (years)		69 ± 10	41–84
Body weight (kg)		63.7 ± 9.9	47.5–89.0
Body mass index (kg/m^2^)		24.1 ± 4.1	19.0–34.3
Serum creatinine (mg/dL)		0.84 ± 0.17	0.57–1.3
Creatinine clearance (mL/min)		73.9 ± 21.7	47.2–126.3
Serum albumin (g/dL)		3.8 ± 0.4	3.3–4.6
Operation time (min)		238 ± 73	140–430

^a^ *lap* laparoendoscopic procedure

**Table 2:** Hypothesis testing for fixed efects model on cefmetazole parameters

Parameter	Fixed effects model	OBJ	- 2 l.l.d.	*p*-value
CL	θ1	491.305		
	θ1 + θ2 × Ccr	476.461	−14.844	0.001
	θ1 + θ2 × 1 / Scr	486.958	−4.347	N.S.
	θ1 × θ2^Gender^ (Gender: male = 1, female = 0)	486.934	−4.371	N.S.
	θ1; Age ≧ 65, θ2; Age < 65	491.291	−0.014	N.S.
	θ1; Alb ≧ 3.8, θ2; Alb < 3.8	491.305	0	N.S.
	θ1 + θ2 × (1 + (4 - stage))	490.054	−1.251	N.S.
	θ1 × θ2^Procedure^	491.074	−0.231	N.S.
	θ1 + θ2 × BW	489.316	−1.989	N.S.
Vd	θ1 × θ2^Gender^ (Gender : male = 1, female = 0)	491.295	0.010	N.S.
	θ1; Age ≧ 65, θ4; Age < 65	490.189	1.116	N.S.
	θ1 + θ2 × BW	473.811	17.494	0.001

-2 l.l.d. : −2 log likelihood difference

*N.S*. Not significant

**Table 3:** Final pharmacokinetic parameter estimates for cefmetazole in patients undergoing colorectal surgery

Pharmacokinetic Parameters	
CLCMZ = θ1 × Ccr	(L/h)
VdCMZ = θ2 × BW	(L)
Estimate	
θ1	0.0704
θ2	0.163
Variability	
ωCL (%)	21.0
ωVd (%)	8.4
σ (%)	13.5

**Fig. 2:**
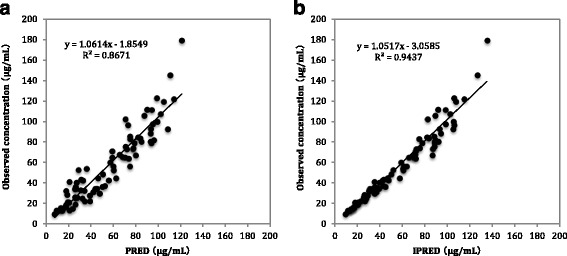
**a** Relationship between observed concentration and predicted concentration based on population mean parameters using the Final model (PRED). **b** Relationship between observed concentration and individual predicted concentration after Bayesian fitting (IPRED)

**Fig. 3:**
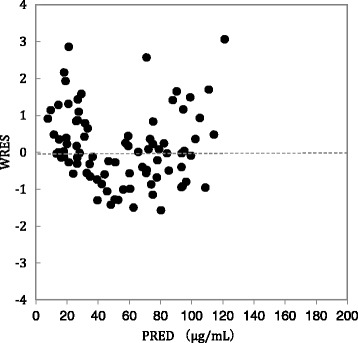
Weighted residuals between observed concentration and PRED (WRES) versus PRED

On regression analysis of Cp and PRED, a correlation coefficient of r^2^ = 0.8671 was obtained (Fig. 2a). On regression analysis of Cp and IPRED, a correlation coefficient of r^2^ = 0.9437 was obtained (Fig. 2b). Weighted residuals (WRES) estimated on the basis of Cp and PRED were almost uniformly distributed within a range of about ± 3 when WRES = 0 (Fig. 3). The results of bootstrap validation of the estimated pharmacokinetic parameters are shown in Table 4. The convergence rate was 100% (200/200).

**Table 4:** Bootstrap validation of the estimated pharmacokinetic parameters

Parameter	Final model^a^ (mean ± S.E.)	Bootstrap^b^ (mean ± S.E.)	Difference^c^
θ1 (CL)	0.0704 ± 0.0029	0.0703 ± 0.0029	−0.001%
θ2 (Vd)	0.163 ± 0.0054	0.164 ± 0.0057	0.6%
ωCL	0.210 ± 0.0137	0.202 ± 0.0350	−3.8%
ωVd	0.084 ± 0.0053	0.070 ± 0.0377	−16.7%
σ	0.135 ± 0.0045	0.133 ± 0.0165	−1.5%

^a^Obtained from the original data set

^b^Calculated from 200 bootstrap replications

^c^{(Bootstrap value - Final model value)/Final model value} × 100

Table 5 shows the probability of attaining predicted serum concentrations above the MIC of *Bacteroides fragilis* : MIC target attainment rate, according to Ccr and Bw, calculated on Monte Carlo simulation of the population pharmacokinetic parameters. The MIC target attainment rate 3 h after the initial dose of CMZ was 3.39 to 15.6% in patients with a Ccr of ≥90 to <130 mL/min, 57.9 to 81.5% in those with a Ccr of ≥50 to <90 mL/min, and 96.0 to 96.7% in those with a Ccr of ≥10 to <50 mL/min. The MIC target attainment rate at 2 h after the initial dose of CMZ was 52.9 to 82.2% in patients with a Ccr of ≥90 to <130 mL/min and 90% or higher in patients with a Ccr of 50 to <90 mL/min, irrespective of Bw. In patients with a Ccr of ≥10 to <50 mL/min, the MIC target attainment rate 5 h after the initial dose of CMZ was 81.2 to 90.6%.

**Table 5:** The target attainment rate above MIC80 of *Bacteroides fragilis* calculated on Monte Carlo simulation

			The target attainment rate			
Bw (kg)	Ccr (mL/min)	2 h	3 h	4 h	5 h	6 h
≧40 to 50	≧90 to <130	52.87%	3.39%	0.00%	0.00%	0.00%
	≧50 to <90	91.24%	57.89%	19.42%	3.45%	0.37%
	≧10 to <50	98.66%	96.67%	92.96%	81.20%	66.08%
≧50 to 60	≧90 to <130	72.44%	8.56%	0.14%	0.00%	0.00%
	≧50 to <90	92.17%	72.40%	33.66%	8.28%	2.33%
	≧10 to <50	98.19%	96.71%	93.43%	88.53%	74.18%
≧60 to 70	≧90 to <130	82.16%	15.57%	1.58%	0.00%	0.00%
	≧50 to <90	92.69%	81.51%	44.25%	15.74%	4.78%
	≧10 to <50	97.27%	96.06%	93.16%	90.56%	79.45%

## Discussion

Outside of Japan, cefoxitin and cefotetan are used as perioperative antimicrobial prophylaxis in patients who undergoing surgery for colorectal cancer [2–4]. Because these drugs cannot be used in Japan, however, CMZ, which is also a cephamycin’s antibiotics, is widely employed. Few studies have examined the optimal dosage of CMZ in patients under surgery, including the intraoperative administration of additional doses. We believe that it is extremely important to assess the optimal dosage of CMZ on the basis of PK/PD theory.

The CL_CMZ_ obtained on population pharmacokinetic analysis was dependent on Ccr, and Vd_CMZ_ was dependent on Bw. These findings were considered reasonable because more than 85% of CMZ is excreted as the unchanged compound in the urine, and excretion is mainly renal. A CL_CMZ_ of 7.04 L/h (Ccr : 100 mL/min) and a Vd_CMZ_ of 10.4 ± 1.6 L (Bw : 47.5 to 89.0 kg) were generally consistent with the results of Borin et al. (CL : 6.96 L/h, Vd : 11.9 ± 4.2 L) [7] and Wong-Beringer et al. (Vd : 0.14 to 0.28 L/kg) [8].

Finally, models were prepared for estimating CL_CMZ_ on the basis of Ccr, and Vd_CMZ_ on the basis of Bw. These data can be obtained from serum chemical analysis before surgery, thus resulting in a clinically appropriate and practical model.

On diagnosis of the final model, regression analysis showed that a high correlation coefficient was obtained between observed serum CMZ concentrations and predicted CMZ concentrations based on population mean parameters, with a high regression coefficient, suggesting that predicted concentrations based on population mean parameters were good. On bootstrap validation, the mean bootstrap values approximated the final model values. The robustness was 100% (200/200) on normal completion of calculation, thus demonstrating the internal validity of the population parameters.

When the optimal treatment schedule for CMZ was assessed using the obtained population pharmacokinetic parameters, the MIC attainment rate at 2 h after initial treatment was 52.9 to 82.2% in patients with a Ccr of ≥90 to <130 mL/min irrespective of Bw. In contrast, the MIC attainment rate was less than 20% at 3 h after initial treatment. This finding suggested that additional doses should be given every 2 h after the initial dose in patients with a Ccr of ≥90 to <130 mL/min. Collaborative guidelines for AMP published in 2013 [4] recommended that additional doses of cefoxitin, a drug belonging to the same category as CMZ, should be given every 2 h after the initial dose in patients with normal renal function. Therefore, the timing for additional doses of CMZ in patients with a Ccr of ≥90 to <130 mL/min is considered consistent with the recommendations of current guidelines [3, 4].

However, the essential goal of AMP is to decrease bacterial counts to a level that does not cause infection, given the susceptibility of the individual patient to infection. Therefore, treatment schedules should be adjusted according to the Bw and renal function of individual patients, rather than indiscriminately giving additional treatment to all patients.

Our results suggest that additional dose of CMZ should be given every 2 h in patients with a Ccr of ≥90 to <130 mL/min, every 3 h to those with a Ccr of ≥50 to <90 mL/min, and every 4 to 5 h in those with a Ccr of ≥10 to <50 mL/min. Our limitation was the low number of renal failure (Ccr of <50 mL/min) patient (*n* = 1). Therefore our recommended dosage should be adjusted to each individual clinical situation and care must be taken with patients to Ccr of <50 mL/min.

Further studies of larger number of patients are required to confirm whether our results are consistent with external data and to assess the relation between the MIC attainment rate and the risk of surgical site infection.

## Conclusions

We studied to determine the optimal dosage of CMZ during surgery in patients with colorectal cancer.

Our results suggest that additional dose of CMZ should be given every 2 h in patients with a Ccr of ≥90 to <130 mL/min, every 3 h to those with a Ccr of ≥50 to <90 mL/min, and every 4 to 5 h in those with a Ccr of ≥10 to <50 mL/min.

## Abbreviations

AMP: Antimicrobial prophylaxis
Bw: Body weight
Ccr: Creatinine clearance
CL: Clearance
CMZ: Cefmetazole
HPLC: High-performance liquid chromatography
MIC: Minimum inhibitory concentration
OBJ: Objective function
PK/PD: Pharmacokinetic/pharmacodynamic
Vd: Volume of distribution
